# *Ascaridia galli* - An old problem that requires new solutions

**DOI:** 10.1016/j.ijpddr.2023.07.003

**Published:** 2023-07-24

**Authors:** Johan Höglund, Gürbüz Daş, Behdad Tarbiat, Peter Geldhof, Désirée S. Jansson, Matthias Gauly

**Affiliations:** a Department of Biomedical Sciences and Veterinary Public Health, Section for Parasitology, Swedish University of Agricultural Sciences, Uppsala, Sweden; b Institute of Nutritional Physiology ‘Oskar Kellner’, Research Institute for Farm Animal Biology (FBN), Wilhelm-Stahl-Allee 2, 18196, Dummerstorf, Germany; c Laboratory for Parasitology, Faculty of Veterinary Medicine, Ghent University, Salisburylaan 133, B-9820, Merelbeke, Belgium; d Department of Clinical Sciences, Swedish University of Agricultural Sciences, Uppsala, Sweden; e Free University of Bolzano, Department of Animal Science, Piazza Università 5, 39100, Bolzano, Italy

**Keywords:** Poultry roundworm, Diagnosis, Control, Targeted treatment, Anthelmintic resistance

## Abstract

Reports of *Ascaridia galli* in laying hens in Europe have increased since the ban on conventional battery cages in 2012. As this parasite is transmitted directly via the faecal-oral route by parasite eggs containing a larva, it is reasonable to assume that the escalating problem is related to the increased exposure now occurring in modern welfare-friendly cage-free housing systems. On many farms, *A. galli* reappears in subsequent flocks, even though the birds have no access to the outdoors, biosecurity is high and empty houses are cleaned and disinfected during downtime. Since the egg production cycle lasts only ≈80 weeks and recombinant antigen production for helminth vaccines has not yet been solved, the development of a vaccine seems to be an unrealistic option. Therefore, disrupting the life cycle of the parasite by other means, including the strategic use of dewormers, appears to be the key to controlling infection. Of concern is that only one class of anthelmintics is licenced for poultry in Europe and that are usually administered indiscriminately through the birds' drinking water and often too late when the parasite is already established. If current calendar-based parasite control strategies are not changed, there is a risk that resistance to anthelmintics may develop, as has already been demonstrated with nematodes in livestock. We insist that treatments can be more effective and the risk of developing drug resistance can be mitigated if we invest in a better understanding of *A. galli* responses to more prudent and judicious use of anthelmintics. This review identifies knowledge gaps and highlights aspects of sustainable parasite control that require further research to support commercial egg producers.

## Highlights

• Research efforts to optimise the control of *Ascaridia galli* in laying hens are needed.
• The use of a TT approach based on a combination of diagnostic tools is a viable control option.
• Investigating the extent to which *A. galli* is resistant to BZ drugs should be a priority.
• Advances in diagnostic parasitology offer the potential to fill knowledge gaps.
• When and how to sample the flocks should not be ignored.

## Graphical abstract

**Figure:**
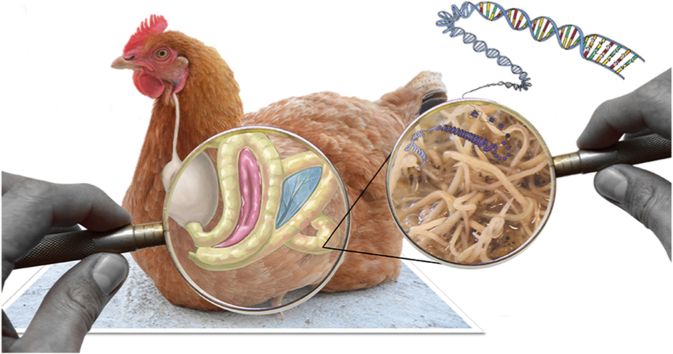

## 1 Background to the problem

More than 30 helminths have been reported from chickens (Shifaw et al., 2021b). Among these, *Ascaridia galli* is the most common parasite, followed by the caecal nematode *Heterakis gallinarum*, which often occurs as a mixed infection. Although *A. galli* has long been known to occur in chickens under a variety of housing conditions worldwide, the parasite was generally less prevalent when laying hens were kept in conventional battery cages separate from their droppings (Permin et al., 1999; Jansson et al., 2010; Sherwin et al., 2013; Wuthijaree et al., 2017; Shifaw et al., 2021b). Due to changes in animal welfare regulations, this type of housing system has been banned in the European Union (EU) since January 2012 (Directive 1999/74/EC). Today, most hens are instead kept in large flocks in housing systems with litter. A similar trend can be observed in several other industrialised countries (Sharma et al., 2019; Shifaw et al., 2023).

The 2000s saw a rapid expansion of free-range laying hens on conventional and organic farms in Sweden (Tarbiat et al., 2020, 2023) as in many other European countries. Although the transition from unfurnished cages to more animal-friendly housing systems has led to an improvement in animal welfare, this has also been the cause of the increased occurrence of *A. galli*. Nevertheless, based on observations in Sweden, where the ban was imposed as early as 1999 and enforced in early 2005, it is not surprising that the change in animal husbandry has also led to an increase in the occurrence of *A. galli* in other industrialised countries (Shifaw et al., 2021b). Especially as there is evidence that some commercial hybrids are more susceptible to infection than others (Gauly et al., 2002; Schou et al., 2003). The parasite is therefore the subject of growing attention and research into sustainable control strategies is of great interest.

In this review, we propose a new approach to the control of *A. galli* on farms based on the principle of targeted treatment (TT) with anthelmintics supported by the use of improved diagnostic methods. As this is an evidence-based intervention strategy, we emphasise the immediate need to evaluate appropriate sampling strategies in combination with diagnostic tools that ideally allow accurate and early detection of infection. Equally important, however, is access to standardised, universally accepted methods for detecting anthelmintic resistance (AR) that can be used in large-scale surveillance. In addition, there are a number of other issues that should be explored (Box 1). All in all, there are several questions that need to be investigated and clarified before this treatment concept can be recommended for practise.

Box 1 **Outstanding questions**

• How widespread is *A. galli* in commercial production systems for laying hens and what are the implications for product performance and efficiency (e.g. feed conversion rate) on a larger scale?
• Which combination of diagnostic tests and sampling strategies is most efficient for the application of TT in commercial production systems for laying hens?
• What are the diagnostic tools' thresholds that should trigger anthelmintic treatment?
• Can a non-invasive egg yolk serology be used to determine when a flock is first infected, and if so, how can this diagnostic approach be used to support TT?
• To what extent are egg producers in a wider geographical context willing to accept the TT approach?
• How will TT influence the number of anthelmintic treatments?
• Can FECRT be used to monitor the efficacy of anthelmintics in large flocks?
• What concentrations are required to reach LD50 after *in vitro* exposure of *A. galli* eggs to BZ in different isolates?
• To what extent does TT influence the risk of developing resistance to anthelmintics?
• What genetic variation exists in *A. galli* and how does this contribute to the pathogenicity and mechanisms associated with the development of resistance to BZ?

Alt-text: Box 1

## 2 Impact

Due to the high fecundity of *A. galli* and the weak immune response of the chicken host, the intensity of infection increases over time (Wongrak et al., 2015). Parasitism is therefore a challenge, especially in intensive production, as more birds per unit area means a greater risk of contamination. The highly resistant *A. galli* eggs excreted in host faeces remain viable under the conditions prevailing in poultry barns and soon develop (embryonate *in ovo*) into eggs with an infective larva (Katakam et al., 2014; Tarbiat et al., 2018; Feyera et al., 2020; Shifaw et al., 2022). More than 88% of *A. galli* eggs deposited in host faeces complete their development after only one to two weeks under optimal laboratory conditions (Tarbiat et al., 2015; Rahimian et al., 2016). Although many ascarid eggs are destroyed within a few months, a small proportion (up to 3%) can survive for up to two years (Thapa et al., 2017), meaning that infectious eggs accumulate in the environment. After ingestion, the larvae hatch from the eggs, and penetrate the intestinal mucosa as part of their development process (Ferdushy et al., 2012). This damages the mucosa and causes irritation and inflammation (Pleidrup et al., 2014). Some larvae soon reappear in the intestinal lumen and become adult worms, which reach sexual maturity after about five weeks and then excrete the parasite's eggs for several months if not expelled (Stehr et al., 2018). When the worm burden is high, this is associated with loss of appetite, diarrhoea and mechanical intestinal obstruction, which in turn can lead to reduced nutrient absorption and depletion of fat reserves in the liver (Daş et al., 2010; Sharma et al., 2018a; Torres et al., 2019; Feyera et al., 2022d). Severe infections have also been shown to reduce weight gain and may even lead to increased mortality (Skallerup et al., 2005; Hinrichsen et al., 2016).

Eventually, the worms may migrate into the oviduct and become lodged in the egg. When such table eggs are discovered during inspection, they are discarded out of concern for consumers (Piergili Fioretti et al., 2005). There is also evidence that *A. galli* may increase the risk of interactions with bacterial infections in chickens, making them more susceptible to bacterial infections with *Escherichia coli* and *Pasteurella multocida* (Dahl et al., 2002; Permin et al., 2006). In addition, concurrent infection with *A*. *galli* and *Salmonella enterica* serovar Enteritidis can lead to increased *Salmonella* excretion (Eigaard et al., 2006). The eggs of the parasite can also serve as a mechanical vector for *Salmonella* (Chadfield et al., 2001).

**Fig. 1:**
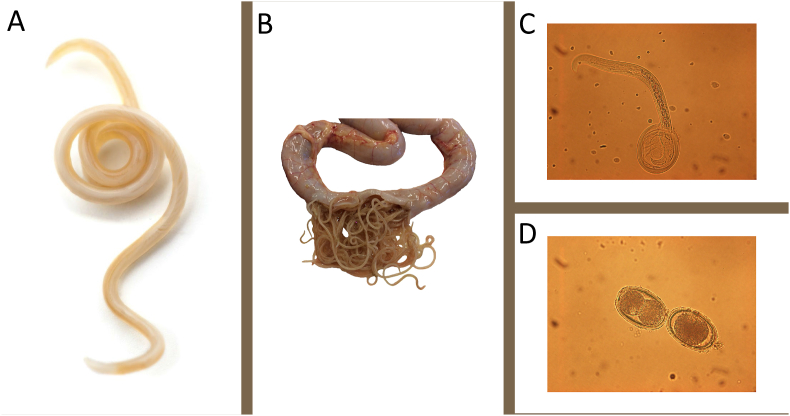
Different developmental stages of *Ascaridia galli* (syn. *A. lineata* and *A. perspillus*). In contrast to most gastrointestinal nematodes in ruminants of interest to veterinary medicine, which are found in clade V, *A. galli* is an ascarid belonging to clade III. The adult parasite (**A**) is a common intestinal parasite in the small intestine of chickens. *A. galli* is the largest nematode in birds, with females growing ≈7–11 cm long. Worm populations usually show an aggregated (skewed) distribution within their host population, with a few hosts harbouring many worms while most hosts have few or no worms. In the case of severe infection (**B**), the intestine becomes obstructed. The life cycle is direct, and birds are infected orally by eggs containing an infective third stage larva (L3). As shown in **C**, these eggs hatch inside the host and the larvae penetrate the intestinal wall where they moult. Although most larvae remain in the mucosa for an extended period, some L4 return to the lumen after about three weeks, reach sexual maturity and begin producing unembryonated eggs 5–8 weeks after infection (**D**). The eggs begin to develop once they are in the external environment, where they can survive for several months even under harsh conditions.

Although many cases of ascaridiosis are mild and do not cause clinical symptoms, there is no doubt that a high rate of infection, as shown in Fig. 1, should be prevented. Surprisingly, few studies have systematically investigated the impact of *A. galli* on commercial egg laying farms in terms of chicken health and production performance. However, there is some evidence that egg production and daily feed intake may be affected (Sharma et al., 2019; Stehr et al., 2019; Tarbiat et al., 2020). It is therefore important to assess the impact in terms of prevention and control of *A. galli*, especially in modern egg production facilities. Prevention includes appropriate husbandry based on high standards of on-farm biosecurity and sanitation of the empty barn before introducing new birds. Generally, additional control measures are also required, such as treatment with anthelmintics, but only if necessary.

## 3 Control and associated risks

One of the most effective methods of controlling ascarids is to treat the birds with an anthelmintic (Tarbiat et al., 2016a; Feyera et al., 2022b). This is sometimes important because even if infection with *A. galli* elicits specific immune responses, the protective role is questionable (see Sharma et al., 2019). All available results indicate that flocks of laying hens on litter usually remain infected to some extent and sometimes even increase with age until the end of the production cycle, which usually lasts up to 80 weeks (e.g. Zloch et al., 2018).

Several drugs, including benzimidazoles (fenbendazole and flubendazole), imidazothiazoles (levamisole and pyrantel) and macrocyclic lactones (ivermectin), have been shown to be effective against *A. galli* (Sharma et al., 1990; Saemi Soudkolaei et al., 2021; Feyera et al., 2022b). However, regardless of the choice of drug, a restrictive, well-considered approach should be taken, as there is a risk of AR development if these drugs are used unwisely. This is because resistance develops as a result of intensive selection through repeated treatments with substances in the same drug class (Abongwa et al., 2017). The lack of efficacy of anthelmintics has been documented for all major drug classes, particularly those used to control strongylids in ruminants and horses as well as some drugs used against ascarids in horses and humans (Krücken et al., 2017; Woodgate et al., 2017; Nielsen, 2022). Although the genetic basis for the development of AR is not always understood, AR is nevertheless associated with mutations that give resistant worms a survival advantage over susceptible worms (Wolstenholme et al., 2004). Thus, when resistant worms are treated, they pass on their genes to a greater extent than the susceptible worms that are eliminated by the treatment and the population gradually becomes more resistant.

It is therefore worrying that only one class of anthelmintics (namely benzimidazoles) is approved for poultry, at least in the EU and the USA. These drugs are often administered via the drinking water over several days to ensure that all hens are exposed to the drug. However, treatment through drinking water carries the risk of underdosing, as intake is voluntary (Tarbiat et al., 2016a; Feyera et al., 2021). The drug is also usually administered at a time when the flock is infected with a large worm population that has high genetic variability and treatments can be repeated up to six times during a production cycle (Höglund and Jansson, 2011; Tarbiat et al., 2023). Taken together, this exposes the parasites to a uniform selection pressure, as it is not possible to switch to other substances with different modes of action. It is known from sheep that a high drenching frequency and underdosing contribute to the development of AR (Falzon et al., 2014). Consequently, if the use of anthelmintics in chickens is not refined, there is a risk of selection for BZ resistant poultry ascarids.

Interestingly, resistance of *Ascaridia dissimilis* to fenbendazole (a BZ compound) has recently been described based on *post-mortem* results from a controlled efficacy study in turkeys in the USA (Collins et al., 2019). Similarly, in another study in which both chickens and turkeys were treated with fenbendazole and/or albendazole, efficacy against all common ascarids (*Ascaridia* spp. and *Heterakis gallinarum*) was below the 90% threshold at which the drugs are considered effective (Yazwinski et al., 2013). Further evidence of the lack of efficacy of fenbendazole and levamisole against adult forms was also provided in a later study at a facility in northwest Arkansas (Yazwinski et al., 2020). Another recent study investigating the *in vivo* efficacy of fenbendazole and levamisole against *A. galli* in chickens also found insufficient activity (Saemi Soudkolaei et al., 2021). Finally, a recent study from Sweden suggests that a similar situation is developing (Tarbiat et al., 2023). Although this was not clearly linked to resistance by the authors, it shows that the efficacy of BZ drugs does not always meet expectations and suggests the possibility of the emergence of AR in poultry ascarids.

Although the faecal egg count reduction test (FECRT) has been used both in experimentally *A. galli* infected chickens (Feyera et al., 2021) or naturally helminth-infected chickens (Feyera et al., 2022c) to assess efficacy of anthelmintics, there are no validated non-invasive protocols for detecting drug resistance in *A. galli* in birds (see 5.2). Confirmation of resistance therefore requires euthanasia of the birds and detection of larvae and adult worms in the intestines, and ideally experimental treatment trials (Yazwinski et al., 2022). Therefore, it is currently difficult to systematically investigate the field situation with regard to how BZ resistance evolves in commercial flocks. Investment in the development of resistance detection methods that can be used on farms with egg laying hens is not only timely but also deserves increased research attention, not least because the resistance status of the parasite needs to be monitored to detect the problem before it manifests clinically. Apart from what can be gained for the egg industry, a deeper understanding of AR in poultry ascarids is also of interest for related ascarids in humans, pigs, horses and companion animals.

## 4 Targeted treatment

The risk of AR is related to a number of factors, such as the intensity, timing and type of anthelmintic use (Falzon et al., 2014). Nevertheless, it is unrealistic to stop deworming completely, not least because there are few scientifically proven alternatives with similar efficacy. However, to slow and mitigate the risk of selection, the use of anthelmintics should be coordinated and demand-driven. This can be achieved by deworming according to the principles of targeted treatment (TT), where the flock is dewormed based on information about the intensity of infection confirmed by a diagnostic test. Rather than treating according to a fixed, calendar-based, blanket treatment plan, TT involves deworming the entire flock based on risks or parameters that quantify the severity of the infection. TT is thus based on the concept that animals have a parasite load above which they become ill and suffer production losses. The flocks are therefore only treated when it is deemed necessary. In ruminants the number of parasite eggs in the faeces can be used to determine when the herd needs treatment, but other indicators can also be used (Kenyon and Jackson, 2012). In this way, TT helps to reduce the overall need and cost of anthelmintics, as treatment is evidence-based and demand-driven.

The TT concept was introduced many years ago and is applied to grazing livestock to avoid worm-related negative impacts on production while maintaining the long-term efficacy of anthelmintics by keeping a pool of unselected parasites in refugia that are not exposed to treatment, thereby reducing the risk of developing drug resistance (Kenyon and Jackson, 2012; Charlier et al., 2014). TT is therefore sustainable because it aims to promote the long-term and prudent use of anthelmintics. Another option would be targeted selective treatment (TST), but this is considered unrealistic for poultry as each flock often consists of thousands of birds. Since the flock is the relevant ”unit” to be diagnosed and treated, only the TT approach is of interest to producers working with laying hens. However, just as with grazing livestock, a trade-off must be made between the risk of developing AR and accelerating the damage caused by the parasite. It appears that many flocks on commercial farms are re-infected with residual eggs from the previous flock, but the timing of reappearance of parasite eggs varies between 6 and 18 weeks after the birds are brought into the egg-laying facility, depending on the effectiveness of cleaning and disinfection during the downtime (Höglund and Jansson, 2011). As the currently available drugs (i.e. BZ, available as oral suspensions or emulsions for administration to chickens via drinking water) have no residual activity, it has also been shown that birds in a contaminated environment become reinfected within a week of the end of treatment (Tarbiat et al., 2016a). Thus, if a flock is dewormed late in the production cycle, this has only a temporary positive effect and thus little influence on the contamination of parasite eggs in the barn and its surrounding areas (Tarbiat et al., 2023). To avoid both production losses and an accumulation of parasite eggs in the laying facility, it therefore seems sensible to monitor the flock with a diagnostic test to determine the optimal times for treatment.

To date few studies have looked at TT programmes to control *A. galli* based on non-invasive diagnostics. Three studies using an arbitrary threshold of 200 eggs per gram (EPG) of faeces as a treatment indicator in the TT groups showed a lower worm burden and a lower cumulative number of eggs in the faeces (Tarbiat et al., 2016b, 2020, 2022). Interestingly, in one of these studies, the hens in the TT group also showed higher egg production as well as better feed conversion and plumage condition compared to the conventionally dewormed groups and the untreated control group (Tarbiat et al., 2020).

**Fig. 2:**
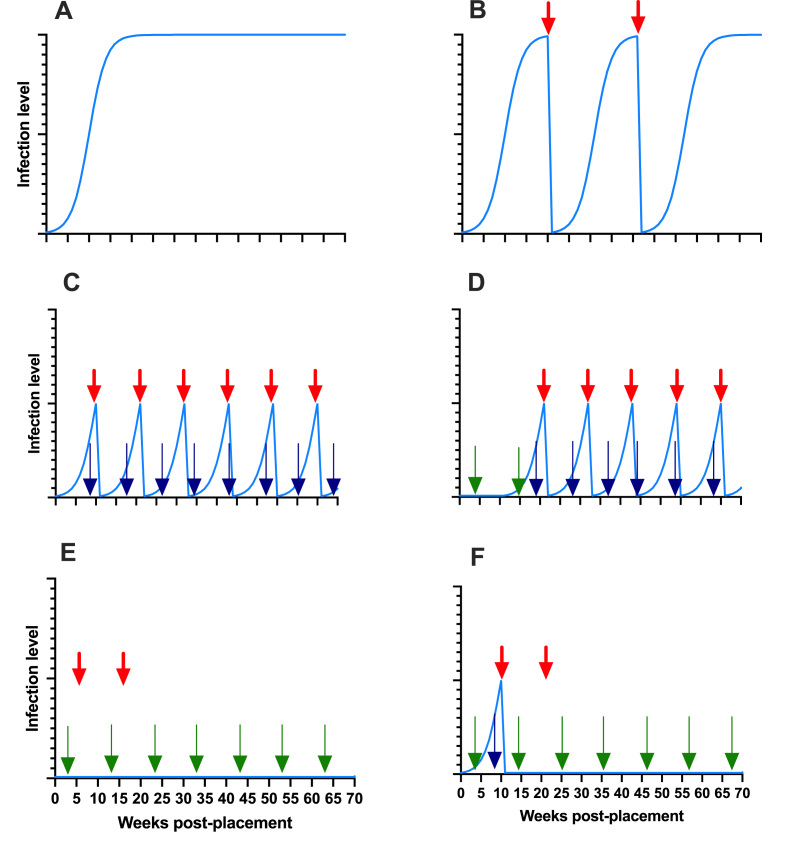
Hypothetical results of some targeted treatment strategies against *A. galli*. The blue line shows population dynamics of the parasite. The red arrow indicates anthelmintic treatments, while the blue and green arrows indicate diagnostics based on coproscopy and detection of antibodies in the yolk, respectively. The diagram in **A** shows the typical sigmoidal pattern of population increase. While there is concern that the TT strategy shown in **C** (based on repeated faecal egg counts, FEC) may lead to more treatments than in **B**, and where anthelmintics are used based on clinical signs, it is not known what the long-term impact would be. Since infection rates in **C** would reduce environmental contamination with parasite eggs in the long term, it is assumed that the risk of re-infection of subsequent flocks would be low. Another possibility is to base surveillance on the initial detection of ascarid antibodies in the yolk and then test with FEC, as shown in **D**. The number of treatments could then be reduced. However, if treatment decisions are based solely on the detection of antibodies, there is a risk that this will lead to strong selection on AR when no parasite eggs are present in refugia, as shown in **E**. This issue may be mitigated to some extent if treatment is postponed until parasite eggs are detected by coprological analysis, as shown in **F**. (For interpretation of the references to colour in this figure legend, the reader is referred to the Web version of this article.)

Although the TT approach seems promising, further testing is needed to evaluate how it works on a larger scale on commercial farms and how effective it is, taking into account both the level of refugia and the number of treatments, which should ideally be kept to a minimum (Fig. 2). As TT primarily requires the use of existing and new diagnostic tools, its usefulness needs to be validated under commercial egg production conditions. In addition, it is important to define appropriate treatment thresholds to optimise the use of the different combinations of non-invasive markers proposed below (see 5.1). Nevertheless, it is of highest priority that the control strategy is effective without selection for AR. This is considered to be ensured if a small proportion of unselected, drug-susceptible parasite eggs remain in refugia in the environment. An important question is therefore what proportion of the parasite population needs to be in refugia to mitigate and delay selection for AR. Therefore, the impact of the proposed TT approaches on the underlying genetic factors causing AR needs to be thoroughly investigated before they can be recommended (Fig. 2). Furthermore, as it is important to achieve a balance between effective parasite control and management of drug resistance, the effectiveness of the proposed control strategy, but also its impact on farm productivity, needs to be investigated before TT can be recommended. Only then will TT be a viable option for farmers.

## 5 Diagnostics

### 5.1 Laboratory diagnosis

The TT approach obviously requires access to a diagnostic tool so that informed evidence-based, infection control decisions can be made. Diagnostics, therefore, play a central role, but the tools must be both cost-effective and reliable. In this context, it is also important to ensure that the sampling strategy is feasible so that meaningful information on the status of the flock can be obtained from the samples collected.

Adult *A. galli* in the small intestine can be detected at *post-mortem*, as they are easily visible to the naked eye when fully grown. It is therefore possible to test the farm for overt patent infection*.* However, it is more difficult for the non-specialist to detect the early stages of *A. galli* and *H. gallinarum*. Due to the tissue-associated phase in the life cycle of *A. galli*, even wet-sieving of intestinal content is unable to detect the presence of early infections (Luna-Olivares et al., 2015). More importantly, basing TT on this detection method seems problematic for several reasons. First, as shown in Fig. 2, the TT concept is based on repeated monitoring of infection status in the flock. At least in Europe, it would generally not be considered ethical to sacrifice birds for diagnostic purposes. Secondly, it is costly, and the likelihood of samples being submitted is low. Thirdly, even if this approach is accepted, there is still controversy about how many hens need to be tested and how they should be selected. In our opinion, it is easier and more ethical for producers to monitor their flocks by submitting samples for testing that have been obtained in a non-invasive way.

There are several coproscopic and thus non-invasive methods for detecting *A. galli* in bird faeces, all based on flotation of the parasite eggs (Table 1). McMaster and Mini-FLOTAC, for example, are the most commonly used methods for quantifying gastrointestinal nematode eggs in livestock, and their diagnostic performance has recently been evaluated for ascarid eggs in chickens (Daş et al., 2020; Shifaw et al., 2021a). Although these methods are similar, the McMaster method is faster and provides better accuracy than Mini-FLOTAC but may suffer from low sensitivity and precision when the intensity of infection is low. In addition, it is difficult to distinguish microscopically between eggs of *A. galli* and *H. gallinarum* which can occur as a mixed infection. However, both problems can be solved with the help of a molecular tool. For example, it was recently shown that digital droplet (dd)PCR is not only more sensitive than semi-quantitative flotation, but also capable of determining the relative abundance of DNA copies of internal transcribed spacer-2 (ITS-2) of *A. galli* and *H. gallinarum* in faecal samples (Tarbiat et al., 2021). In addition, a loop-mediated isothermal amplification in conjunction with a lateral flow dipstick (LAMP-LFD) assay that recognises the ITS-2 was recently presented for the visual detection of *A. galli* eggs in faecal samples (Panich et al., 2023). Although the performance of these tools has never been compared such as in *Haemonchus contortus*, it is reasonable to assume that ddPCR is the most sensitive molecular assay (Ljungström et al., 2018; Baltrušis and Höglund, 2023). However, a general problem with molecular tools is the cost. On the other hand, these are likely to decrease as they become part of routine diagnostics and are integrated into highly automated platforms.

**Table 1:** A relative comparison of diagnostic tools for the detection and quantification of Ascaridia galli infections in chickens at individual and flock level^a^.

Sample-base	Necropsy^b^	Coproscopy			Serology		Molecular tools	
Method	Section of intestine	McMaster	Mini-FLOTAC	Copro-ELISA	ELISA		ddPCR	LAMP-LFD
Target	Adult and juvenile worm stages	Worm eggs	Worm eggs	Worm antigen	Plasma IgY	Egg-yolk IgY	Worm DNA	Worm DNA
Qualitative diagnostic performance^c^	+++	++	+++	+++	+++	+++	NI	NI
Correlation to adult worms^d^	(+++)	++	++	+(+)	+	+	NI	NI
Correlation to larvae	(+++)	0	0	++	+++	+++	NI	NI
Early diagnosis^e^	+++	0	0	++(+)	+++	+++	0	0
Species-specificity^f^	Yes	0	0	No	No	No	Yes	Yes
Invasiveness^g^	Yes	No	No	No	Yes	No	No	No
Simultaneous sample analysis	+	++	++	+++	+++	+++	+++	+++
Performance speed	+	+++	+++	++	++	++	+	+
Ethical concern	+++	0	0	0	+	0	0	0
Sample handling and storage	++	+++	+++	+++	+++	+++	+	+
Cost^h^	+++	+	++	++	++	++	+++	+++

**0**: non-existing; **+**: low; **++**: moderate; **+++**; high: **NI** not investigated yet.

a The comparisons of methods are based on the best knowledge and experience of the authors, but may nevertheless be subjective.

b This includes both wet-sieving to recover adult and larval stages from the intestinal lumen and tissue digestion to recover larvae.

c It is based on the assessment of diagnostic sensitivity, specificity, positive and negative predictive values and likelihood ratios or accuracy through ROC analysis.

d Implies the correlation between adult worm counts or worm size or infection dose to measured variable.

e Refers to the ability of a method to detect infections before the worm has maturated, i.e. lays eggs.

f Relates to the distinction between *Ascaridia galli* and *Heterakis gallinarum*.

g Refers to the need to cull or bleed birds for sampling.

h Approximate expenses of analysis per sample, based on sample material (e.g. chicken vs. faeces), the time required by a qualified technician and the consumables needed.

An alternative approach is the use of a species-specific coproantigen assay (Oladosu et al., 2022). Since antigens released by all developmental stages are detected, it has the advantage of being able to detect the presence of parasites even when eggs are not collected by flotation (e.g. in the case of early infection with immature worms or intermittent shedding of eggs). Indeed, when the diagnostic performance of coproantigen ELISA in the course of infections recently was compared with other methods at different weeks post-infection, it was found that infection rates can be followed with high accuracy and repeatability using this method (Oladosu et al., 2023). However, regardless of the method used, further work is needed to investigate how representative the results of a pooled sample are for large commercial farms, although it has recently been shown that pooled fresh faecal samples using egg counts would be sufficient to give an indication of infection levels (Shifaw et al., 2021a). In addition, there are data suggesting that exposure to *A. galli* is similar regardless of location in the barn (Yazwinski et al., 2020). Still, practical strategies for on-farm sampling remain challenging and undoubtedly require further investigation.

Alternatives to faeces as sample material include the collection of serum and yolk to measure parasite-specific IgY antibodies using serological methods such as ELISA (Martín-Pacho et al., 2005; Daş et al., 2017; Sharma et al., 2018b; Dao et al., 2019). Although antibody levels in yolk are lower than in serum, the advantage of using yolk over serum is that sampling is non-invasive and there are reliable cut-off values (Daş et al., 2018). It has been shown experimentally that IgY antibodies can be detected two weeks after inoculation of birds with infectious *A. galli* eggs and it appears that antibody transfer from blood to yolk is very rapid (Rahimian et al., 2017). Thus, this serological test can detect infection before the parasite reaches patency and eggs appear in the faeces (Schwarz et al., 2011; Stehr et al., 2019). A possible disadvantage is that cross-reactivity between *A. galli* and *H. gallinarum* occurs (Daş et al., 2017). However, if seroconversion is monitored from the beginning of the production cycle, this tool can be used as an initial screening tool to find out when the flock becomes infected. The results can thus serve as a warning signal that intervention may be needed at a later stage (Fig. 2).

### 5.2 Assessment of anthelmintic efficacy

In the most recent guidelines for evaluating the efficacy of anthelmintics in poultry, information derived from faecal egg counts (and hence FECRT) before and after treatment is considered to support, but not to replace, *post-mortem* worm count data (Yazwinski et al., 2022). This is particularly important because a standardised FECRT protocol is essential not only for the validity and reproducibility of efficacy results, but also for the availability of fully comparable data across studies and regions worldwide. For example, among other factors, route of medication used (i.e. oral administration vs. administration in-water) has a significant impact on worm and egg counts (Feyera et al., 2021). This is an important source of variation that needs to be defined and controlled in a validated standard FECRT protocol. Nevertheless, there is also an urgent need for the development and validation of practically feasible diagnostic methods (both biological and molecular) for several other reasons.

The FECRT was originally developed in Australia and focused on strongylid nematodes in sheep. It has been common practise for several decades to classify the *in vivo* effect of drugs in a range of naturally infected domestic animals (Coles et al., 2006). The calculation of effect is based on the number of parasite eggs before and, depending on the substance, 7–21 days after treatment. If the estimated reduction of faecal eggs is below 95% and with a lower confidence interval of 90%, the parasite isolate is considered resistant. According to the original design, at least ten animals per anthelmintic should be included and the baseline for these animals should be above 200 EPG. Although the procedures and statistical framework for performing and interpreting a FECRT have been revised over the years (Wang et al., 2017; Denwood et al., 2023) and new guidelines for ruminants, horses and pigs have recently been published (Kaplan et al., 2023), the test has not yet been scientifically evaluated for this purpose in chickens. To this end, a standardised protocol is needed, both in terms of the number of birds included in the test and the number of parasite eggs counted, in order to obtain comparable data in studies in all parts of the world. In this context, it is important to consider how to deal with confounding factors, such as the influence of coprophagy, worm expulsion, diurnal variation and crowding effects on parasite egg production, which may affect FECRT results (Wongrak et al., 2015; Stehr et al., 2018; Heckendorn et al., 2009). It may be possible to identify the same birds before and after treatment and to include an untreated control group if the flocks are small and individual birds can be identified by appropriate wing or leg marking, so that repeatedly tested birds can be included in the statistical evaluations. However, for pragmatic reasons, it is unrealistic to routinely test the same birds before and after treatment when studying large flocks of chickens in commercial floor housing systems. Therefore, careful consideration must be given to how these aspects can be managed before applying FECRT to birds.

A complementary tool to the FECRT is the use of various bioassays such as the Egg Hatch Assay (EHA), the Larval Migration Inhibition Assay (LMIA) and the Larval Developmental Assay (LDA). In these assays, unembryonated parasite eggs are exposed to gradually increasing concentrations of the anthelmintic *in vitro* to determine the optimal concentration that will act on the parasites. Depending on the endpoint, these methods rely on the ability of the parasites to embryonate, develop or migrate in response to increasing drug concentrations. However, these assays have been developed and validated primarily for the evaluation of strongylid resistance in ruminants and horses (e.g. Demeler et al., 2012, 2010). Although versions of these types of assays have also been used with *A. galli*, the number of studies is quite limited (Tarbiat et al., 2017; Feyera et al., 2021, 2022a). For the future, tests with different *A. galli* isolates with defined resistance status need to be performed to establish LD50 reference values, especially in response to BZ-drug exposure (Box 1).

In addition, molecular PCR methods have been used to investigate genetic changes associated with resistance (Wolstenholme et al., 2004). Although there are some genetic studies on *A. galli*, they mainly focus on other aspects such as taxonomic and population genetic issues (Katakam et al., 2010; Höglund et al., 2012; Urbanowicz et al., 2018). This contrasts with the situation in strongyles, where the genetic basis for resistance in several clade V nematodes has been intensively studied, especially in the major species. In comparison, knowledge about the genetic characteristics of ascarids, which unlike strongylids belong to the clade III, is still in its infancy. For example, in *H. contortus*, one of the best-studied strongylids, the genomic regions responsible for resistance to all major drug classes have recently been identified (Doyle et al., 2022). This study has also confirmed that resistance to BZ is related to point mutations in one of the beta-tubulin genes previously studied with advanced genomic tools (Baltrušis et al., 2018, 2020). However, mutations at the same sites have repeatedly been shown not to be involved in the corresponding evolution of resistance *in A. galli* as well as in ascarids of other animals (Tarbiat et al., 2017). This suggests that *A. galli* reacts genetically differently from strongyles or interferes with isotypes that have not yet been identified. Although the discovery of transcriptional differences between BZ -treated and untreated *A. galli* has identified alternative potential markers (Martis et al., 2017), further research on this topic has been temporarily halted. As a result, there is a lack of genetic tools to assess BZ-resistance that can be used in epidemiological studies or routine diagnostics.

In summary, apart from *in vivo* tests, there is a lack of standardised and validated methods for detecting AR in *A. galli*. Alternatives are unfortunately still not available or suitable as routine screening tools for chicken ascarids. Therefore, it is difficult to conduct systematic studies to investigate the extent of the problem uniformly and on a large scale. This is unfortunate because it is important that detection methods are available before AR becomes a widespread clinical problem. The development and evaluation of such methods is therefore urgently needed and must be intensified.

## 6 Concluding remarks

*A. galli* is on the rise and is now increasingly found in laying hens kept for commercial egg production. The parasite thus appears to be an escalating problem that requires the increased attention of researchers, who still have much to learn about improving control strategies. First and foremost, efforts need to be made to develop and validate evidence-based control approaches that take advantage of diagnostics and combine them with the use of anthelmintics, without ignoring the risk of resistance development. While we can learn a lot from how dewormers are used for sustainable control of strongylids in ruminants and horses, we need to be aware that *A. galli* belongs to a different clade. Knowledge of the genetics of *A. galli* in response to drug selection is still in its infancy, and there are no universally accepted methods yet for monitoring the AR status of ascarids without killing birds. In addition, the management of laying hens in current floor-based systems is very different from the management of grazing animals. For example, an all-in/all-out approach is practised, individual diagnoses, selective treatments and grazing management cannot be readily applied to laying hens. Our knowledge of controlling *A. galli* with anthelmintics therefore still has many gaps and poses some challenges, some of which are addressed in this article (Box 2). Nevertheless, the use of a TT strategy, in which diagnostics play an important role, could be a viable option for the future, hopefully providing more effective treatment than today and theoretically reducing the risk of AR.

Box 2 **Research priorities for future research**

• Validation of sampling strategies for the collection of diagnostic data.
• Evaluation of diagnostic tools when used in parallel with TT.
• Multicentre field trials of the efficacy of treatment with TT.
• Development of biological and molecular tools to detect AR.
• Accurate surveys of prevalence and impact in laying hens across Europe.

Alt-text: Box 2

## Declaration of competing interest

The authors declare that they have no competing interests.
